# Herpes Simplex Virus (HSV-1) Encephalitis Mimicking Glioblastoma: Case Report and Review of the Literature

**DOI:** 10.3390/jcm3041392

**Published:** 2014-12-12

**Authors:** Burke A. Cunha, Daniel Talmasov, James J. Connolly

**Affiliations:** 1 Infectious Disease Division, Winthrop-University Hospital, Mineola, NY 11501, USA; E-Mails: dtalmasov@winthrop.org (D.T.); jconnolly@winthrop.org (J.J.C.); 2 State University of New York School of Medicine, Stony Brook, NY 11794, USA

**Keywords:** anti-viral therapy of glioblastomas, CNS viruses, mimics of HSV, encephalitis

## Abstract

Glioblastoma multiforme (GBM) often presents as a brain mass with encephalitis. In a patient with GBM, subsequent presentation with new onset encephalitis may be due to another GBM or Herpes simplex virus 1 (HSV-1) encephalitis. We present a case of HSV-1 encephalitis mimicking GBM in a patient with previous GBM.

## 1. Introduction

Reports exist of the co-occurrence of Herpes simplex virus-1 (HSV-1) encephalitis (HSE) and glioblastoma, of HSE following glioblastoma, and of glioblastoma multiforme (GBM) mimicking HSE [1,2,3,4,5,6,7]. For these reasons patients with a history of glioblastoma presenting with new-onset encephalitis and new focal lesions on imaging are often thought to have a multicentric GBM. Since multifocal and multicentric glioblastomas (GBM) have been reported, HSE may be easily overlooked as a diagnostic possibility, as the clinical presentation of HSE may mimic GBM [8]. We report a case of HSE mimicking GBM in a patient previously diagnosed with GBM.

## 2. Case Presentation

**Figure 1:**
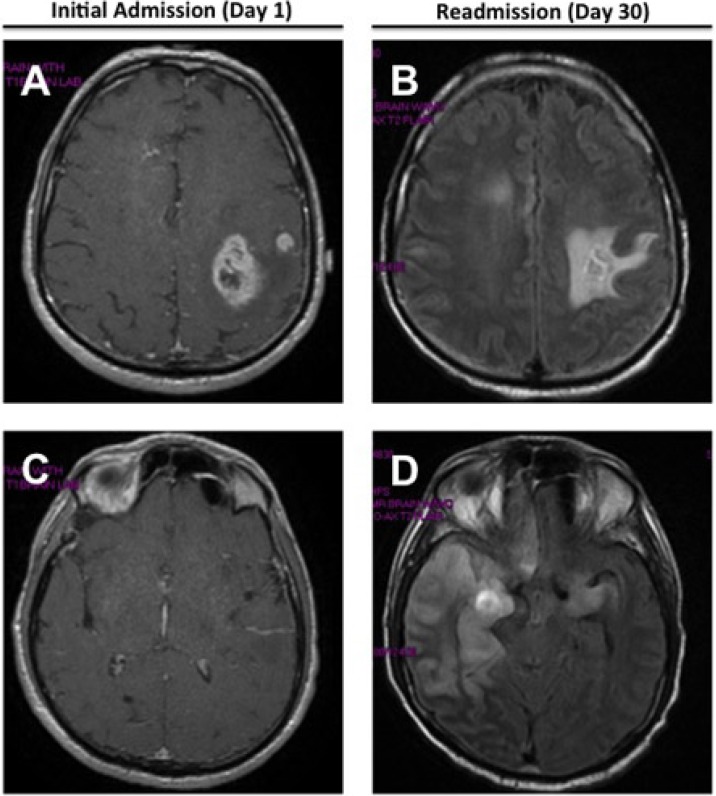
Axial MR images of brain with contrast demonstrating T1 weighted left temporoparietal enhancing lesions on initial presentation (**A**,**C**), and T2 FLAIR right frontotemporal signal abnormalities on hospital readmission (**B**,**D**).

A 60 year old man presented to the hospital with fever and confusion. His history of present illness included an admission one month earlier for slurred speech, right hemiparesis, and a 4.5 kg unintentional weight loss over four months. On that admission, he was afebrile and physical exam revealed dysmetria and decreased strength, sensation, and proprioception of the right upper extremity. Routine admission laboratory tests were within normal limits. Brain magnetic resonance imaging (MRI) demonstrated multiple left temporoparietal enhancing lesions with cerebral edema and mass effect (Figure 1A,C). A left frontal lobe brain biopsy revealed a diagnosis of glioblastoma (WHO grade IV). He was discharged and received three weeks of radiation and chemotherapy with temozolomide. The patient’s past medical history was significant for diabetes mellitus.

On current admission, his vital signs included a temperature of 39.7 °C (103.4 °F) and a pulse of 88/min. Physical examination was unremarkable except for left facial weakness and decreased right upper and lower extremity strength. Laboratory tests included a white blood cell (WBC) count of 0.4 K/mm^3^ (absolute neutrophil count = 0.2 K/mm^3^), a hemoglobin of 12.3 g/dL, and a platelet count of 20 K/mm^3^. The remainder of his routine admission laboratory tests were unremarkable. Chest X-ray (CXR) showed a possible right lower lobe infiltrate. CT scan of the head demonstrated left frontal lobe abnormalities and corpus callosum abnormalities in the left frontoparietal region unchanged from the prior MRI. The patient was given a platelet transfusion, started on meropenem for febrile neutropenia, and tapered off dexamethasone (which he presented on 4 mg twice daily).

The patient underwent a lumbar puncture (LP) after his platelet level increased. Cerebrospinal fluid (CSF) analysis showed 0 WBCs/mm^3^ (nl <5 WBC/mm^3^), 0 RBCs/mm^3^, total protein of 100 mg/dL (nl <50 mg/dL), glucose of 33 mg/dL, LDH of 38 IU/L, and a negative Gram stain. His mental status continued to worsen. Electroencephalography (EEG) demonstrated generalized background slowing with left frontotemporal low amplitude sharp waves. The patient was treated with voriconazole 280 mg (IV) q12h and acyclovir 700 mg (IV) q8h while awaiting CSF culture and viral polymerase chain reaction (PCR) results. Repeat brain MRI with contrast showed a new signal abnormality of the right temporal lobe (Figure 1D). Five days after admission, qualitative CSF PCR was reported positive for HSV-1, confirmed on hospital day 16 by repeat CSF PCR testing. Serum serology was positive for HSV-1 IgG, and negative for HSV-1 IgM and HSV-1 DNA. In the CSF, HSV-1 IgG was positive while HSV-1 IgM was negative, suggesting reactivation. The patient required intubation and blood transfusions. After a week, a repeat EEG showed global slowing, but no frontotemporal sharp waves. His hospital course was complicated by nosocomial pneumonia. Despite granulocyte colony-stimulating factor (G-CSF), his profound neutropenia did not improve and he later succumbed from multi-organ failure.

## 3. Discussion

In four reported cases of glioblastomas initially diagnosed as HSE, patients showed dramatic short-term symptomatic improvement following treatment with acyclovir. One of these cases had concurrent steroids and acyclovir, but three other cases reported improvement following administration of acyclovir alone [5,6,7]. The response to acyclovir raises the possibility that, at least in some cases, glioblastomas may be caused or complicated by otherwise subclinical HSV-1. Alternately latent HSV-1 central nervous system (CNS) infections may be aggravated by the emergence of a new malignancy, or latent HSV-1 CNS infection may cause glioblastoma. Here we present a case of HSV-1 encephalitis mimicking GBM.

In our case, initial CSF fluid cytology and serology were not diagnostic for HSE, but CSF PCR was positive for HSV-1. With HSE, lymphocytic pleocytosis, normal glucose, and increased protein are typical CSF findings, however HSE can present with normal CSF analysis [9,10,11]. The missing pleocytosis in our case is likely due to the neutropenic state of the patient. Detection of HSV DNA via CSF PCR in HSE cases may be negative during the first 24–48 h following symptoms, and is less sensitive by 5–7 days following antiviral treatment [12]. CSF cytological/serological findings in HSE are moreover dependent on the proximity of the intraparenchymal infection to the meninges.

The MRI findings in our case were suggestive of HSV-1 encephalitis, which has a predilection for temporofrontal lobes, and commonly involvement of the insular and cingulate cortices [9,13]. Interestingly, the propensity to infect certain brain regions and brain cell types, referred to as neurotropism, has been used to engineer HSV-1 to preferentially infect and destroy malignant glioma cells, although the neurotropic mechanisms are still not well understood [14,15,16,17]. It is notable that most reported cases of HSE in patients with glioblastoma multiforme are due to HSV-1. Only one case of encephalitis secondary to Herpes simplex virus-2 (HSV-2) infection has been reported in a patient receiving corticosteroids following subtotal resection of a temporal glioblastoma (Table 1) [18].

**Table 1:** Clinical and laboratory features of glioblastoma multiforme (GBM) cases complicated by herpes simplex virus-1 (HSV-1) encephalitis (HSE).

Case	Reference	Demographics	Seizures Accompanying Encephalitis	HSE Preceding GBM	HSE with GBM	HSE Following GBM	CSF Cell Count	CSF Chemistry	CSF HSV-1 PCR	MRI/CT	EEG	GBM Response to Acyclovir
1	Rees and Howard, 1999	41F	+	2 years			<5 WBC/mm^3^ 0 RBC/mm^3^	Normal	NA	High signal left medial temporal lobe	NA	+
2	Rees and Howard, 1999	49M	+		GBM mimicking HSE		<5 WBC/mm^3^ 0 RBC/mm^3^	Normal	−	Diffuse high signal change right anterior temporal lobe	NA	+
3	Rees and Howard, 1999	72F	−		GBM mimicking HSE		<5 WBC/mm^3^ 0 RBC/mm^3^	Normal	−	Extensive left medial temporal high signal, some abnormality medially in right temporal lobe	NA	−
4	Kleffmann *et al.*, 2012	60F	+		GBM mimicking HSE		NA	Prot = 41 mg/dL	− HSV antibodies	Medial temporal lesions with spread to dorsal thalamus	NA	NA
5	Kleffmann *et al.*, 2012	65M	+		GBM mimicking HSE		<5 WBC/mm^3^ 0 RBC/mm^3^	Prot = 82 mg/dL Lactate = 1.8 mg/dL	−	Left fronto-temporal lesion expansion	Left frontal focus	NA
6	Kleffmann *et al.*, 2012	55M	+		GBM mimicking HSE		<5 WBC/mm^3^ 0 RBC/mm^3^	Prot = 65 mg/dL Lactate = 3.0 mg/dL	−	Left occipital lesion expansion	NA	−
7	Mateen *et al.*, 2014	79F	+			Subtotal resection 10 days prior	64 WBC/mm^3^ 2739 RBC/mm^3^	Prot = 179 mg/dL Gluc = 39 mg/dL	*	Bilateral abnormal (right > left) temporal, insular, and orbitofrontal cortices	NA	NA
8	Lins *et al.*, 2004	61M	+			4–6 months	52 WBC/mm^3^ 0 RBC/mm^3^	Albumin = 776 mg/L Lactate = 4.1 mmol/L	+	Right fronto-temporal lobe hypodensity	NA	−
9	Sheleg *et al.*, 2001	28F	−		+		NA	Normal	+ post-mortem tissue PCR	Right temporal lobe enhancement	NA	NA
10	Nam *et al.*, 2011	70M	−		GBM mimicking HSE		9 WBC/mm^3^ 0 RBC/mm^3^	Prot = 45 mg/dL Gluc = 65 mg/dL	−	Hyperintense lesion without enhancement left medial temporal lobe	Slowing on left temporal derivations/focal or lateralized sharp and/or slow wave complexes over left temporal derivations	+
11	Smithson and Larner, 2013	48M	+		GBM mimicking HSE		<5 WBC/mm^3^ 0 RBC/mm^3^	Normal	−	Edematous change confined to right anterior and medial temporal lobes	NA	+
12	Schiff and Rosenblum, 1998	24F	+			6 months	9 WBC/mm^3^ 2210 RBC/mm^3^	Prot = 28 mg/dL Gluc = 52 mg/dL	NA	Decreased size of left hemisphere tumor with moderate ventricular dilation	Left hemisphere spikes, background slowing	NA
13	Cunha *et al.*, 2014	60M	−			1 month	<5 WBC/mm^3^ 0 RBC/mm^3^	Prot = 100 mg/dL Gluc = 33 mg/dL Lactate = 38 IU/L	+	Multiple left temporo-parietal enhancing lesions; subsequent signal abnormality of the right temporal lobe	Generalized background slowing with left frontotemporal low amplitude sharp waves	Transient

NA = not available; GBM = glioblastoma multiforme; HSV = herpes simplex virus; * Only case of HSV-2 encephalitis.

The initial differential diagnoses of our case also included bacterial superinfection and fulminant tumor progression. No autopsy was performed to definitively exclude these possibilities. Multiple spinal taps were performed throughout the hospitalization and were consistently negative for bacterial culture and gram stain, fungal culture, and cryptococcal antigen. In view of the negative CSF bacterial cultures and the more likely diagnosis of HSV-1 encephalitis, bacterial superinfection and fulminant tumor progression are less probable.

Bilaterality of HSE is not rare, however viral antigen is often more abundant on one side [19,20]. In comparison, a neurosurgical study of glioma localization reported in 209 patients with gliomas showed that 72.2% are solitary tumors, whereas 27.8% presented in multiple sites [8].

Immunologic studies have found that adults with GBMs have a higher rate of IgG seropositivity for both HSV-1 and cytomegalovirus (CMV), another herpetic virus, than the general population [21]. Further studies in patients with GBMs detected CMV at a high rate (80%) in serum using PCR analysis, and in tumor tissue using multiple assays (>90%) [22,23]. Given the high reported incidence of CMV in patients with GBMs, HSV-1 may also be present in GBM cases.

The lack of therapeutic response to acyclovir in our case is most plausibly attributable to the delayed diagnosis and concomitant immunocompromised state. Even with appropriate diagnosis and treatment of HSE, mortality remains high [24]. Viral cultures from the patient’s CSF were not obtained to test for acyclovir resistance. The possibility of an acyclovir-resistance HSV-1 strain therefore cannot be excluded, however acyclovir resistance is uncommon in the facility where the patient presented.

Anecdotal reports of acyclovir responses in GBM cases mistaken for HSE raise the question of whether anti-HSV therapy may play an adjunctive role in GBM treatment [5,6,7]. Acyclovir has been shown to inhibit growth of glioblastoma cell lines *in vitro*, as well as inhibit regulatory T-cells that support glioblastoma progression through immunosuppressive effects [25,26]. Given these results, additional studies are warranted to further assess HSV-1 involvement in GBM and the efficacy of anti-HSV drugs as an adjuvant treatment.

There are a number of potential explanations for the co-occurrence of HSE in patients with GBM. In a patient such as ours, HSE may be due to a new HSV-1 infection in the setting of preexisting GBM, or may be a result of reactivation of latent HSV-1 due to steroid therapy. A study of trigeminal ganglion cells from HSV-1 latently infected mice revealed that dexamethasone administration induced viral reactivation in a dose-dependent manner [27]. A similar conclusion was reached in rabbits, where dexamethasone in combination with an immunosuppressive drug caused reactivation [28]. HSV-1 encephalitis has also been reported in patients taking natalizumab for multiple sclerosis, and patients taking tumor necrosis factor-alpha inhibitors [29,30]. Suppression of intracellular killing by CD8^+^ cytotoxic T-cells by steroid and immunomodulatory therapy may potentiate reactivation of HSV-1 in the brain.

The relationship of HSE and GBM is complex, with potential mimics of HSE presenting as GBM, or the possibility of concomitant HSE occurring in cases of GBM (as in our case). Open questions remain regarding the relationship of HSV-1 to the etiology of GBM, and of GBM following HSE since HSV may mimic GBM and GBM may mimic HSV. If a patient with preexisting diagnosis of GBM presents with new encephalitis and focal lesion on brain imaging, both multifocal GBM and HSV-1 encephalitis should be considered as potential diagnoses.

Clinicians should be aware of many guises of GBM and HSV, particularly their propensity to occur in the same patient, *i.e.*, HSE may closely mimic GBM. For this reason, we conclude CSF HSV PCR should be included in all cases of GBM presenting with encephalitis, particularly those cases with previous GBM.

## 4. Conclusions

Herpes simplex virus 1 (HSV-1) in normal hosts may present as aseptic meningitis, meningoencephalitis, or encephalitis. HSV-1 is the “great imitator” of several central nervous system (CNS) infections and disorders. HSV-1 may mimic CNS infections, e.g., *Listeria monocytogenes* and some CNS infections may mimic HSV-1 encephalitis, e.g., glioblastoma multiforme (GBM). We present a case of HSV-1 encephalitis mimicking GBM and review the literature. Interestingly, our literature review revealed there have been reports of GBM partially responding to acyclovir in cases of GBM thought to be HSV-1. This suggests that HSV-1 may have a role in the etiology of oncogenic transformation of GBM. The literature review raised several important questions. Does HSV-1 encephalitis predispose to GBM later in life? Is GBM caused by HSV-1? Should GBM be treated with acyclovir in addition to the usual therapy? Clearly, our case report and the others in the literature should prompt research into the role of HSV-1 in GBM.
